# Gallstones and cholecystectomy in relation to risk of intra- and extrahepatic cholangiocarcinoma

**DOI:** 10.1038/bjc.2011.607

**Published:** 2012-01-12

**Authors:** H Nordenstedt, F Mattsson, H El-Serag, J Lagergren

**Affiliations:** 1 Upper Gastrointestinal Research, Department of Molecular Medicine and Surgery, Karolinska Institutet, Stockholm, Sweden; 2 Michael E. DeBakey Veterans Administration Medical Center and Baylor College of Medicine, Houston Center for Quality of Care and Utilization Studies, Houston, TX, USA; 3 King's College London, Division of Cancer Studies, London, UK

**Keywords:** bile duct cancer, biliary tumour, cohort study, gallstones, population-based

## Abstract

**Background:**

Cholangiocarcinomas are highly lethal tumours of the intrahepatic or extrahepatic biliary tract. The aetiology is largely unknown, and the potential roles of gallstones and gall bladder removal (cholecystectomy) need to be addressed in a large study with a long follow-up.

**Methods:**

A population-based nationwide Swedish cohort study was carried out, in which patients hospitalised for gallstone diagnosis with or without gallbladder removal (cholecystectomy) between 1965 and 2008 were identified in the Swedish Patient Registry. The cohort was followed up for cancer in the Swedish Cancer Registry. The observed numbers of intra- and extrahepatic cholangiocarcinomas that developed after one year of follow-up were compared with the expected numbers, calculated from the corresponding background population, and the relative risks were estimated by standardised incidence ratios (SIRs) and 95% confidence intervals (CIs).

**Results:**

Among the 192 960 non-cholecystectomised individuals with gallstones, there was a more than two-fold overall increased risk of both intra- and extra- hepatic cholangiocarcinomas, which remained stable over the follow-up period (SIR 2.77, 95% CI 2.17–3.49, and SIR 2.58, 95% CI 2.21–3.00, respectively). In the cholecystectomy cohort, including 345 251 people and 4 854 969 person-years, 325 incident cholangiocarcinomas were identified, of which 98 (30%) were intrahepatic and 227 (70%) were extrahepatic. Initially (1–4 years after surgery), the risk was increased for both intrahepatic cholangiocarcinoma (SIR 1.80, 95% CI 1.19–2.62) and extrahepatic cholangiocarcinoma (SIR 2.29, 95% CI 1.83–2.82), but no increase remained after 10 years of follow-up or more (SIR 1.10, 95% CI 0.79–1.48, and SIR 0.87, 95% CI 0.70–1.07, respectively).

**Interpretation:**

Gallstones seem to increase the risk of both intra- and extrahepatic cholangiocarcinoma. However, this risk seems to decline to the level of the background population with time after cholecystectomy.

Cholangiocarcinomas are highly malignant tumours that can arise anywhere in the biliary tract. They are usually described as either intra- or extrahepatic, according to their anatomical location. For reasons that are unknown, the incidence of intrahepatic cholangiocarcinomas in the United States has nearly tripled during the past decades, whereas the incidence of extrahepatic cholangiocarcinomas has stayed steady (Shaib and El-Serag, 2004). Cholangiocarcinoma, the second most common type of primary liver cancer after hepatocellular carcinoma (Shaib and El-Serag, 2004) has a worse prognosis than virtually all tumours (5-year survival is lower than 5%) (McLean and Patel, 2006). The aetiology is largely unknown, but predisposing conditions include primary sclerosing cholangitis (Charatcharoenwitthaya *et al*, 2008), diabetes and obesity. In addition, intrahepatic stones (hepatolithiasis), bile duct adenoma or papilloma, choledochal cysts, and possibly tobacco smoking (only in association with primary sclerosing cholangitis) and hepatitis C could be involved in some cases (Welzel *et al*, 2007b). Some of these conditions are thought to increase the risk of cholangiocarcinoma by inducing chronic inflammation or increasing bile duct pressure.

Gallstones might induce biliary inflammation, and cholecystectomy is typically followed by dilation of the bile ducts (Chung *et al*, 1990), which might also cause inflammation and thereby possibly increase the risk of cholangiocarcinoma. Previous studies addressing gallstones or cholecystectomy in relation to extrahepatic cholangiocarcinoma have revealed contradictory results, and there are virtually no valid studies that have investigated the potential associations with intrahepatic cholangiocarcinoma. Therefore, we assessed the intra- and extrahepatic cholangiocarcinoma risk in patients with gallstones who did and did not undergo cholecystectomy in a large population-based cohort study with a long and complete follow-up.

## Materials and Methods

### Study design

A detailed description of the study design was published elsewhere (in a study of the risk of oesophageal cancer) (Freedman *et al*, 2001), but in the current study the cohorts were substantially extended. Two cohorts of patients with gallstone disease were identified in the nationwide Swedish Patient Registry during the period 1965–2008. The gallstone cohort included patients who had not undergone cholecystectomy, while the second cohort included patients who had undergone cholecystectomy. The study participants were identified in the patient registry through unique personal identity numbers, that are assigned to all Swedish residents, containing information on date of birth and sex (Ludvigsson *et al*, 2009). With almost complete coverage, the patient registry contains information on the majority of discharges from inpatient care between 1965 and 1986 and information on every discharge since 1987 and on (National Board of Health and Welfare, 2005b). For each hospitalisation, up to eight discharge diagnoses coded were registered, according to the Swedish version of the International Classification of Diseases (the 7th version for 1964–1968, the 8th version for 1969–1986, the 9th version until 1996 and the 10th version thereafter). Up to six surgical codes were also assigned, according to the Swedish Classification of Operations and Major Procedures. Validation surveys suggest that almost 90% of registered diagnoses are correct when compared with the medical files. As private inpatient care in Sweden was negligible during the relevant time for this study, hospital-provided medical services were, in effect, population-based. For the cholecystectomy cohort, we identified all records between 1965 and 2008 that contained a cholecystectomy code (‘Swedish Classification of Surgical Procedures’ edition 6: 5350, 5351, 5352, 5356, 5357, or 5359; and edition 7: JKA20 or JKA21) and a diagnosis of gallstones, and for the gallstone cohort we identified all records with a diagnosis of gallstones, but no code for cholecystectomy during the study period. Entry into either cohort was recorded as the date of discharge following the first recorded hospitalisation with a cholecystectomy code or a gallstone code in the patient registry. The regional ethical committee in Stockholm approved the study.

### Follow-up

Information on dates of deaths and emigrations were obtained from the Total Population Registry, a complete and updated registry, which has a maximum two weeks’ delay in reporting. Information on cancer was obtained through linkage with the Swedish Cancer Registry, which was established in 1958 to provide a complete cancer database for clinical and epidemiological research purposes. All clinicians and pathologists in Sweden are required to report all cancer cases to the cancer registry, and validation studies have shown a completeness rate of 98% (Mattsson *et al*, 1985). Virtually all cancer cases (99%) in the registry have been morphologically verified (National Board of Health and Welfare, 2005a). The cancer registry contains data on date of cancer diagnosis, codes for specific sites and histology of cancers, and hospital codes. The cancer registry provided data on prevalent cancer cases at entry into the cohort and incident cancers diagnosed during follow-up. Follow-up continued until diagnosis of any cancer (excluding non-melanoma skin tumours), emigration, death, or end of the observational period (December 31st 2008). We only evaluated first and primary cancers.

### Statistical analyses

The Swedish Cancer Registry provided a file with all diagnosed cancer cases categorised according to ICD7 for the entire period of the study. The expected number of cases was calculated by multiplying the observed number of person-years by age-, sex-, and calendar-specific nationwide cholangiocarcinoma incidence rates. Standardised incidence ratios (SIR), that is, the ratio of the observed to the expected number of cases, were used as the measure of relative risk. All person-time and cancers identified during the first year of follow-up were excluded to allow a minimum latency interval between exposure and outcome and to avoid detection bias, that is, earlier detection of prevalent cancer cases identified only because of the cholecystectomy or the gallstone disease. Outcome measures were estimated by calculating SIRs for intrahepatic cholangiocarcinoma (ICD7 code 155.0; histopathology code 076) and extrahepatic cholangiocarcinoma (ICD7 codes 155.2, 155.3, 155.8, 155.9; histopathology code 096), and their 95% confidence intervals (CI), assuming that the number of cases followed a Poisson distribution. To avoid tumour misclassification, for example, by liver metastases or hepatocellular cancer, we only included cases with a verified histopathology code representing primary cholangiocarcinoma.

## Results

### The gallstone cohort

After exclusion of the first year after entry, 192 960 people were included in the gallstone cohort and the median duration of follow-up was 6.4 years, thus providing 1 440 848 person-years at risk. Some characteristics of the cohort members are presented in Table 1. Women constituted about 60% of this cohort and the median age at entry was 68.1 years. There were 241 incident cholangiocarcinoma cases observed during the follow-up, and out of these 72 (30%) were intrahepatic and 169 (70%) were extrahepatic.

### The cholecystectomy cohort

The cholecystectomy cohort included 345 251 individuals, and the median duration of follow-up was 11.9 years, resulting in 4 854 969 person-years at risk. Characteristics are presented in Table 1. There were about twice as many women as men in the cohort, and the women were in general younger at entry into the cohort (50.4 years compared with 57.8 years for men, median value). Among 325 incident cholangiocarcinomas registered during the follow-up period, 98 (30%) were intrahepatic and 227 (70%) were extrahepatic.

### Risk of intra- and extrahepatic cholangiocarcinoma in the gallstone cohort

As presented in Table 2, the 72 incident cases of intrahepatic cholangiocarcinoma identified in the gallstone cohort, were more than were expected in both men (SIR 3.13; 95% CI 2.14–4.42) and women (SIR 2.54, 95% CI 1.81–3.46) compared with the background population. After 10 years or more, the risk remained higher (SIR 2.38, 95% CI 1.43–3.71). The risk was highest in those under 60 years of age at the onset of follow-up, and then decreased with age (Table 2).

There were 169 incident cases of extrahepatic cholangiocarcinoma registered during the follow-up, resulting in a more than two-fold overall increased risk compared with the background population (SIR 2.58, 95% CI 2.21–3.00) (Table 2). The more than two-fold increased risk remained after at least 10 years of observation (SIR 2.12, 95% CI 1.53–2.85). The risk was similar in men and women and was fairly stable irrespective of age at diagnosis (Table 2).

### Risk of intra- and extrahepatic cholangiocarcinoma in the cholecystectomy cohort

The 98 new cases of intrahepatic cholangiocarcinoma identified during follow-up in the cholecystectomy cohort were more than expected, compared with the corresponding background population (SIR 1.38, 95% CI 1.12–1.69) (Table 2). However, the risk decreased with time after cholecystectomy, and among the 43 cases who were followed up 10 years or more after cholecystectomy, no statistically significantly increased risk remained (SIR 1.10, 95% CI 0.79–1.48). The pattern was similar for extrahepatic cholangiocarcinoma. During the follow-up period there were 227 new cases of this tumour among persons who had undergone cholecystectomy. This resulted in an increased overall risk compared with the background population (SIR 1.25, 95% CI 1.09–1.42), but the risk decreased with time. It was strongest in the early period (1–4 years after surgery; SIR 2.29, 95% CI 1.83–2.82), but among the 89 cases who were followed up 10 years or more after cholecystectomy, no significant increase remained (SIR 0.87, 95% CI 0.70–1.07) (Table 2). There were no substantial differences in SIR between age groups, but the risk was higher in males compared with females for both types of cholangiocarcinoma (Table 2).

### Sensitivity analyses

The results remained virtually unchanged after stratification according to anatomical site of the extrahepatic tumours. When excluding all individuals with a recorded pre-existing condition representing diabetes, obesity or sclerosing cholangitis (14 242 and 10 278 individuals in the gallstone cohort and the cholecystectomy cohort respectively), the results were unchanged (data not shown).

## Discussion

This cohort study of patients with gallstones with or without cholecystectomy revealed an increased risk of both intra- and extrahepatic cholangiocarcinoma among patients with gallstones. However, this risk decreased with time after cholecystectomy and returned to the level of the background population after 10 years.

The findings of a decreasing risk of intra- and extrahepatic cholangiocarcinoma with time after cholecystectomy, and a remaining increased risk among non-cholecystectomised gallstone patients in the present study suggests that gallstones are a risk factor for these tumours. On the other hand, cholecystectomy *per se* does not seem to decrease the risk of cholangiocarcinoma as was previously reported in a smaller cohort study from Sweden, including 23 cases of extrahepatic cholangiocarcinoma (Ekbom *et al*, 1993). According to the present study, cholecystectomy rather seems to bring the increased risk caused by the gallstones back to the level of the background population.

Methodological advantages of the study include the population-based design, the large number of incident cholangiocarcinomas, the complete and long follow-up, and the separate consideration of intrahepatic and extrahepatic cholangiocarcinomas. Confounding by known risk factors, that is, primarily obesity, diabetes, and sclerosing cholangitis, might be a problem in this observational study (Diehl, 1991; Welzel *et al*, 2007a). However, this source of error was addressed by a sensitivity analysis for both cohorts, excluding those with a recorded, pre-existing diagnosis of diabetes, obesity and sclerosing cholangitis, and our results remained virtually unchanged.

Nearly all diagnoses of gallstone disease in Sweden are radiologically verified, typically by abdominal ultrasound, resulting in a high specificity for this diagnosis. However, many gallstone patients experience no or mild symptoms, and are thus not hospitalised. Patients with gallstones in our study are therefore likely to have more severe gallstone disease, making it difficult to generalise the results to the average individual with gallstones. On the other hand, if a large proportion of the general population has asymptomatic gallstones, our risk estimates regarding gallstones are likely to be underestimated as the background population was used for comparison. Furthermore, patients hospitalised for gallstones and cholecystectomy might have been more likely to have co-morbidity than those not hospitalised; SIR may not have completely adjusted for these differences and therefore we cannot exclude residual confounding. The indication for cholecystectomy in the present cohort was related to gallstones. In Sweden, the proportion of men who are cholecystectomised for a gallstone complication (i.e., cholecystitis, pancreatitis or jaundice) is similar to the proportion of men who are cholecystectomised due to pain. In women, more than two out of every three cholecystectomies are performed on an indication of pain (GallRiks, 2008). This indicates that a higher proportion of the gallstones that were removed in men might have resulted in a complication, with ensuing inflammation and cholestasis, compared with the seemingly more uncomplicated gallstones that were removed in women. A true protective effect of cholecystectomy in gallstone patients with complication-prone gallstones might therefore be less visible in women, yielding an attenuated SIR. Interestingly, even though age at diagnosis was the same in both the gallstone and cholecystectomy cohorts, the cholecystectomy patients tended to be up to 15 years younger at entry into the cohort. Hypothetically, this could be because the cancer development is delayed in patients who have had gallstones for several years before cholecystectomy.

Previous literature is sparse and not conclusive regarding the risk of intra- or extrahepatic cholangiocarcinomas in gallstone patients or cholecystectomised patients. In a nationwide case-control study from Denmark, cholecystectomy slightly increased the risk estimates for intrahepatic cholangiocarcinoma (OR 1.3 during the first year and OR 1.6 after more than one year), but the number of cases was low (*n*=7), and the results were not statistically significant (Welzel *et al*, 2007b). Another population-based cohort study from Denmark investigated the risk of extrahepatic cholangiocarcinoma in hospitalised patients with gallstone disease or cholecystectomy (Chow *et al*, 1999), and reported a slightly increased risk of extrahepatic cholangiocarcinoma in cholecystectomised patients (SIR 1.6). However, the SIR dropped to below unity five years after surgery, and none of the estimates were statistically significant. In a recent small hospital-based case-control study from China, the risk of both intra- and extrahepatic cholangiocarcinomas was increased in patients who had cholecystectomy, compared with healthy relatives. (Tao *et al*, 2010) Moreover, the occurrence of gallstones increased the risk slightly for extrahepatic but not for intrahepatic cholangiocarcinomas. Finally, a population-based ecological study from the United States found no correlation between the increased annual number of cholecystectomies after the introduction of laparoscopic cholecystectomy, and the incidence of extrahepatic cholangiocarcinoma (Urbach *et al*, 2001). The diverging findings of previous studies are probably explained by low numbers of cholangiocarcinoma cases, which could introduce chance findings, misclassification of extra- and intrahepatic tumours, short follow-up time, and differences in study design.

The findings of the present study may be explained by the effects of chronic inflammation. During the past decade inflammation has become an accepted carcinogenic mechanism in several types of cancer, including cholangiocarcinoma (Komori *et al*, 2008). When the biliary tree is damaged by chronic inflammation, the physiological response is attempted repair through cholangiocyte proliferation (Tavoloni and Schaffner, 1985). Accordingly, proliferation is present in most liver diseases as a consequence of chronic inflammation, particularly when associated with obstructive cholestasis (Xia *et al*, 2007). This is evident in primary sclerosing cholangitis, a chronic cholestatic liver disease that leads to a progressive destruction of intra- and extra- hepatic bile ducts and an increased risk of cholangiocarcinoma, and choledochal cysts (Bergquist *et al*, 2002). The presence of gallstones might increase the expression of proinflammatory cytokines via chronic cholestasis, which would decrease after cholecystectomy.

In conclusion, this large, population-based cohort study with complete follow-up of up to 43 years indicates that gallstones increase the risk of both intra- and extrahepatic cholangiocarcinoma, while the risk of these tumours is reduced back to the level of the background population with time after cholecystectomy.

## Figures and Tables

**Table 1:** Characteristics of the gallstone cohort (consisting of patients with a gallstone diagnosis who did not undergo cholecystectomy in Sweden) and characteristics of the cholecystectomy cohort (consisting of patients who underwent cholecystectomy) in Sweden between 1965 and 2008

	**Gallstone cohort**			**Cholecystectomy cohort**		
	**Men**	**Women**	**Total**	**Men**	**Women**	**Total**
No. of individuals (%)	75 116 (38.9)	117 844 (61.1)	192 960	114 898 (33.3)	230 353 (66.7)	345 251
Number of person-years at risk	498 080	942 767	1 440 848	1 418 031	3 436 938	4 854 969
Median age at entry	69.7	66.6	68.1	57.8	50.4	53.2
Average year of entry	1992	1992	1992	1988	1988	1988
Median follow-up time in years (interquartile range)	5.6 (2.2–12.0)	7.0 (2.7–13.8)	6.4 (2.5–13.2)	10.5 (4.9–20.2)	13.2 (6.0–24.8)	11.9 (5.6–23.3)
Number of cases with intrahepatic cholangiocarcinoma (%)	32 (45.7)	40 (54.3)	72	50 (51.0)	48 (49.0)	98
Median age at diagnosis	70.2	75.9	72.0	71.2	70.6	71.2
Number of cases with extrahepatic cholangiocarcinoma (%)	75 (44.4)	94 (55.6)	169	94 (41.4)	133 (58.6)	227
Median age at diagnosis	75.1	75.6	75.1	72.0	71.9	71.9

**Table 2:** SIRs and 95% CIs of intra- and extrahepatic cholangiocarcinomas in the gallstone cohort, (consisting of 192 960 patients with gallstone disease who did not undergo cholecystectomy) and in the cholecystectomy cohort, (consisting of 345 251 cholecystectomised patients) in Sweden between 1965 and 2008

	**Gallstone cohort**				**Cholecystectomy cohort**			
	**Intrahepatic cholangiocarcinoma**		**Extrahepatic cholangiocarcinoma**		**Intrahepatic cholangiocarcinoma**		**Extrahepatic cholangiocarcinoma**	
	**Cases (no.)**	SIR* (95% CI^†^)	**Cases (no.)**	SIR* (95% CI^†^)	**Cases (no.)**	SIR* (95% CI^†^)	**Cases (no.)**	SIR* (95% CI^†^)
All	72	2.77 (2.17–3.49)	169	2.58 (2.21–3.00)	98	1.38 (1.12–1.69)	227	1.25 (1.09–1.42)
Male	32	3.13 (2.14–4.42)	75	2.88 (2.27–3.61)	50	1.92 (1.42–2.53)	94	1.44 (1.17–1.77)
Female	40	2.54 (1.81–3.46)	94	2.38 (1.92–2.91)	48	1.07 (0.79–1.42)	133	1.14 (0.95–1.35)
								
*Age at cohort entry*								
<60 years	29	4.48 (3.00–6.43)	43	2.78 (2.02–3.75)	49	1.38 (1.02–1.82)	100	1.10 (0.89–1.33)
60–69 years	15	2.41 (1.35–3.98)	45	2.74 (2.00–3.67)	27	1.30 (0.86–1.89)	63	1.17 (0.90–1.49)
⩾70 years	28	2.11 (1.40–3.05)	81	2.41 (1.91–2.99)	22	1.52 (0.95–2.30)	64	1.74 (1.34–2.22)
								
*Years of follow-up*								
1–4	37	3.59 (2.52–4.94)	96	3.71 (3.00–4.53)	27	1.80 (1.19–2.62)	86	2.29 (1.83–2.82)
5–9	16	2.09 (1.19–3.39)	30	1.55 (1.05–2.22)	28	1.68 (1.12–2.43)	52	1.22 (0.91–1.61)
⩾10	19	2.38 (1.43–3.71)	43	2.12 (1.53–2.85)	43	1.10 (0.79–1.48)	89	0.87 (0.70–1.07)

Abbreviations: CI=confidence interval; SIR=standardised incidence ratio.
